# Biofilm-Forming *Enterobacter* sp. W5 Mitigates Cadmium and Polystyrene Microplastic Stress in Wheat via Synergistic Immobilization and Proteomic Reprogramming

**DOI:** 10.3390/plants15111698

**Published:** 2026-05-30

**Authors:** Jiexun Wang, Yun Li, Hao Zhang, Wenxia Wang, Lunguang Yao, Randa S. Makar, Zhaojin Chen, Hui Han

**Affiliations:** 1 Henan Key Laboratory of Ecological Security for Water Source Region of Mid-line of South-to-North Diversion Project, College of Water Resources and Modern Agriculture, Nanyang Normal University, Nanyang 473061, China; wangjiexun0416@163.com (J.W.); ly0210252026@163.com (Y.L.); zhanghao660@nynu.edu.cn (H.Z.); wenxiawang@nynu.edu.cn (W.W.); zhaojin_chen@163.com (Z.C.); 2 Soils and Water Use Department, Agricultural and Biological Research Institute, National Research Centre, Dokki, Cairo 12622, Egypt; lunguangyao@nynu.edu.cn (L.Y.); randa_sgmm@yahoo.com (R.S.M.); 3 Henan International Joint Laboratory of Soil Health and Water Security, Nanyang Normal University, Nanyang 473061, China

**Keywords:** biofilm-forming bacteria, polystyrene, Cadmium, wheat, proteomic

## Abstract

Cadmium (Cd) and polystyrene (PS) microplastic co-contamination in agricultural soils poses a potential threat to food security. Some functional microorganisms in soil can alleviate the dual stress of Cd and PS on crops. In this study, a biofilm-forming bacterium, Enterobacter sp. W5, was isolated from heavy metal-contaminated rhizosphere soil. Strain W5 exhibited Cd removal efficiency (46.3%) and strong biofilm-forming capacity (OD_570_ = 5.05), and it effectively colonized PS microplastic surfaces. XPS analysis detected bacterial functional groups (C–O–C, C=O) and PS-associated signals (O–C=O), which may act synergistically in Cd^2+^ adsorption. Furthermore, XPS and XRD analyses revealed the presence of Cd-containing precipitates (including CdS, CdO, and Cd_3_(PO_4_)_2_). In hydroponic wheat experiments, W5 inoculation alleviated Cd-PS combined stress, thus significantly promoting plant growth and reducing Cd accumulation by 22.6% in roots and by 34.2% in aboveground tissues. Subcellular distribution analysis revealed that W5 enhanced Cd retention in root cell walls, thereby limiting its translocation to active cellular compartments. Proteomic analysis identified a set of 11 consistently downregulated proteins, including A0A3B6HQ68 and A0A3B6KJV9, which were enriched in secondary metabolite biosynthesis pathways. Bioinformatic analysis suggests that these proteins may be associated with Cd stress responses, though their exact roles remain to be verified. Collectively, this study provides a valuable microbial resource and mechanistic insights into the application of biofilm-forming bacteria for mitigating combined heavy metal–microplastic pollution in agricultural systems.

## 1. Introduction

Under the dual drivers of rapid industrialization and profound transformations in agricultural practices, the issue of combined soil pollution in farmland has become increasingly prominent [1]. Cadmium (Cd) exhibits a significant bioaccumulative effect, and it is recognized as one of the most mobile and highly toxic heavy metals in the environment. It primarily enters agricultural ecosystems through mining activities, phosphorus fertilizer application, and atmospheric deposition, subsequently posing serious threats to human health via food chain transfer [2,3]. As a typical microplastic widely present in the environment, polystyrene (PS) can adsorb and enrich heavy metal ions through its carrier effect, thereby altering the migration behavior and bioavailability of Cd in soil [4]. PS was chosen as a model microplastic due to its environmental prevalence, strong Cd adsorption capacity, and comparability with previous studies. Wheat, a major global food crop, has a high capacity for Cd accumulation. While Gan [5] reported that the presence of PS promotes Cd uptake and translocation in wheat, Zong [6] found that PS reduces Cd accumulation in wheat seedlings. These discrepancies may arise from differences in PS concentration, particle size, surface functionalization, and experimental conditions (e.g., hydroponics vs. soil). However, the mechanism underlying Cd toxicity in wheat mediated by PS remains poorly understood.

Microbial remediation technology has emerged as a promising strategy for heavy metal pollution control due to its environmental compatibility and cost-effectiveness [7,8]. Among metal-resistant microbes, bacteria of the genus Enterobacter have attracted particular attention for their capacity to secrete extracellular polymeric substances (EPS) and form biofilm [9]. EPS are rich in functional groups such as carboxyl, amino, and phosphoryl moieties, which can effectively immobilize heavy metal ions through complexation, ion exchange, and precipitation mechanisms [10]. Wang [11] demonstrated that a co-culture of the H_2_S-producing *Enterobacter* sp. A11 and the biofilm-forming Comamonas sp. A23 synergistically removed Cd^2+^ from solution via precipitation, achieving a removal efficiency of 98.7%. However, in the presence of microplastics, the efficacy of biofilm-mediated remediation becomes more intricate. On one hand, microplastics themselves can serve as vectors for heavy metal adsorption. Joseph [12] reported that the biodegradable microplastic PBS exhibits a high adsorption capacity for Cd^2+^, with adsorption behavior conforming to the Freundlich model. On the other hand, microplastic surfaces provide colonization sites for microorganisms, facilitating the formation of a microplastic-biofilm-heavy metal ternary complex. Wang [13] further revealed that the presence of biofilms significantly enhances the adsorption capacity of microplastics for heavy metals, an effect even surpassing that of UV-induced aging. These findings suggest the existence of complex synergistic interactions among biofilms, microplastics, and heavy metals in Cd-PS co-contaminated systems, yet the underlying mechanisms remain poorly understood.

Proteomics technologies serve as a powerful approach for elucidating the molecular mechanisms underlying plant responses to environmental stresses, including pathogen infection, heavy metal exposure, and high temperature [14,15]. In the field of heavy metal stress research, Mittra [16] demonstrated through proteomic analysis that glutathione (GSH) enhances Cd tolerance in Brassica napus by activating the antioxidant system, strengthening sulfur assimilation pathways, and promoting Cd detoxification processes. Regarding microplastic stress, Pehlivan and Gedik [17] employed proteomics to reveal that microplastic exposure modulates the transcriptional expression of functional protein families involved in redox homeostasis, energy metabolism, DNA synthesis, and cell division/repair in Arabidopsis. However, systematic proteomic investigations focusing on the biofilm-forming bacterium–plant–Cd-PS combined pollution system remain scarce. In particular, the mechanisms by which biofilm-producing strains regulate plant protein expression to influence Cd translocation and accumulation are still poorly understood.

Therefore, in this study, rhizosphere soil was collected from heavy metal-contaminated farmland, and biofilm-producing bacteria were screened using biofilm-inducing culture medium. The objectives of this study were to (1) investigate the effect and mechanism of Cd uptake by biofilm-producing bacteria mediated by microplastics through batch adsorption experiments; (2) analyze the mechanisms via which the bacteria influence Cd accumulation, cell wall retention capacity, and intracellular Cd migration in wheat using hydroponic cultivation; and (3) elucidate the molecular mechanisms underlying the bacterial regulation of wheat responses to combined Cd-PS stress through proteomic analysis. The findings are expected to provide a theoretical basis for the application of biofilm-producing bacteria in heavy metal pollution remediation and for ensuring the safety of agricultural food production.

## 2. Results

### 2.1. Screening of Strain W5 and Its Cd Immobilization and Biofilm-Forming Capacities

**Figure 1:**
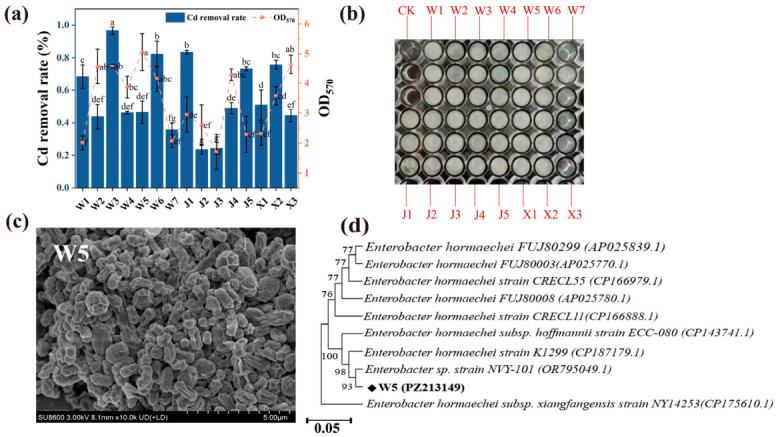
Screening of strain W5. (**a**) Cd immobilization and biofilm-forming capacities of 15 isolated strains; (**b**) Phenotypic image of biofilms; (**c**) Colony morphology of strain W5; (**d**) Phylogenetic tree constructed based on 16S rDNA gene sequences. Bootstrap support values (%, 1000 replicates) are shown at nodes (values <50% are not displayed). The scale bar (0.05) indicates genetic distance. GenBank accession numbers are given in parentheses. Error bars represent standard error (*n* = 3). Different lowercase letters (a–g) for the same test indicator indicate significant (*p* < 0.05) differences between treatments according to Tukey’s test.

A total of 15 bacterial strains capable of stable biofilm formation were isolated from heavy metal-contaminated rhizosphere soil using biofilm screening medium. These strains exhibited Cd removal rates ranging from 23.7% to 96.6%, with strain W5 showing a removal rate of 46.3%. An assessment of biofilm-forming ability revealed OD_570_ values between 1.71 and 5.05, among which strain W5 demonstrated the highest biofilm-forming capacity (OD_570_ = 5.05) (Figure 1a). Compared to the control (CK), all isolates exhibited surface wrinkling and could produce biofilms (Figure 1b). Based on its superior biofilm-forming ability and moderate but stable Cd immobilization capacity (46.3%), together with its strong tolerance to combined Cd-PS stress and plant growth-promoting traits (IAA production and siderophore secretion), strain W5 was selected as the test strain for subsequent investigations (Table S1). The colony morphology of this strain appeared smooth and was accompanied by evident biofilm formation (Figure 1c). Although the 16S rDNA similarity is below the typical 97% threshold for genus-level assignment, phylogenetic analysis and physiological characteristics support the placement of strain W5 within the genus Enterobacter. Therefore, the strain is tentatively identified as Enterobacter sp. W5, pending genome-based confirmation (Figure 1d). The accession number of strain W5 was PZ213149.

### 2.2. Biological Characteristics of Strain W5

Strain W5 produced indole-3-acetic acid (IAA) at 65.4 mg L^−1^ and secreted siderophores with an activity level of ++++ (indicating extremely strong siderophore production, as defined in Table S2); which is higher than that of most other isolates tested (Tables S1 and S2). These traits indicate its plant growth-promoting potential. This strain exhibited a Cd tolerance concentration of up to 800 mg L^−1^ and concurrently showed resistance to gentamicin and tetracycline. Based on Gram staining results, W5 was identified as a Gram-negative bacterium (Table S1). These findings suggest that strain W5 possesses tolerance to heavy metals and resistance to gentamicin and tetracycline, which may contribute to its survival in contaminated environments.

### 2.3. Growth and Cd Removal of Strain W5 in the Cd—PS Composite System

**Figure 2:**
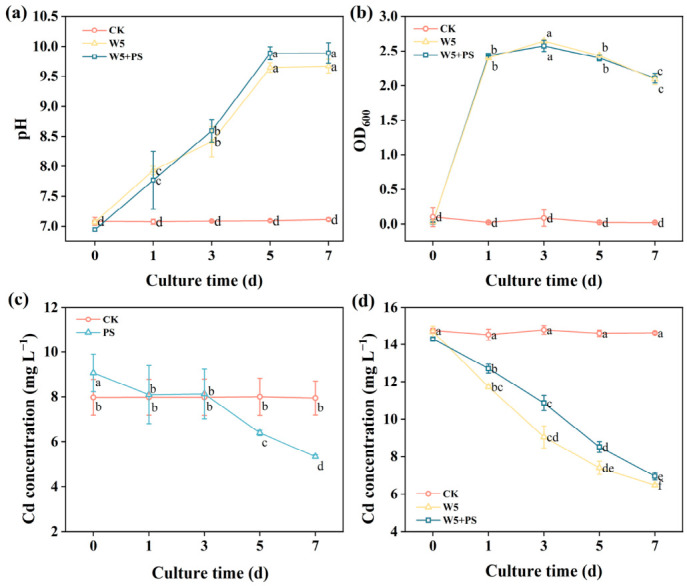
Effects of strain W5 and PS on Cd concentration in the culture medium. (**a**) Changes in pH of the culture medium; (**b**) Bacterial growth (OD_600_); (**c**) Residual Cd concentration in solution after incubation with polystyrene (mg L^−1^); (**d**) Residual Cd concentration in solution after incubation with strain W5 alone and with strain W5 combined with polystyrene (mg L^−1^). Error bars represent standard error (*n* = 3). Bars indicated by the same letter were not significantly (*p* > 0.05) different according to Tukey’s test.

Strain W5 demonstrated good growth adaptability in both the Cd-only contamination system (15 mg L^−1^) and the Cd-PS co-contamination system (Cd 15 mg L^−1^, PS 0.2%, *w*/*w*). When cultured under Cd and Cd-PS stress for up to 7 days, the pH of the culture medium increased to 9.67 and 9.89, respectively. This suggests that strain W5 may secrete alkaline metabolites, which could contribute to a gradual rise in the system pH over time (Figure 2a). Under different treatments, the growth curve (OD_600_) of strain W5 exhibited a trend of an initial increase followed by a decrease, indicating its physiological regulatory capacity in response to Cd stress and Cd-PS co-stress (Figure 2b). In the treatment group containing PS, Cd concentration decreased continuously over the cultivation period, reaching a removal rate of 41% by day 7, thereby suggesting that PS exhibited a certain adsorption effect on Cd (Figure 2c). Under Cd stress (15 mg L^−1^), the Cd removal rate for strain W5 reached 55.9% on day 7. Under Cd-PS composite contamination conditions, the strain maintained stable Cd removal performance, with a removal rate of 51.3% on day 7 (Figure 2d).

### 2.4. Microscopic Mechanisms of Strain W5 Adhesion to PS Surfaces and Cd Adsorption

**Figure 3:**
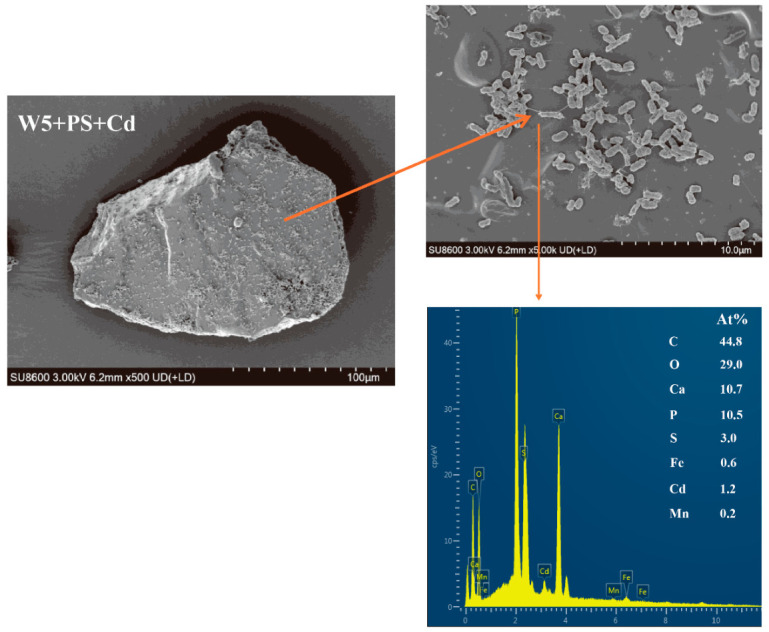
SEM-EDS images revealing the adhesion of strain W5 to PS surfaces and its adsorption of Cd.

**Figure 4:**
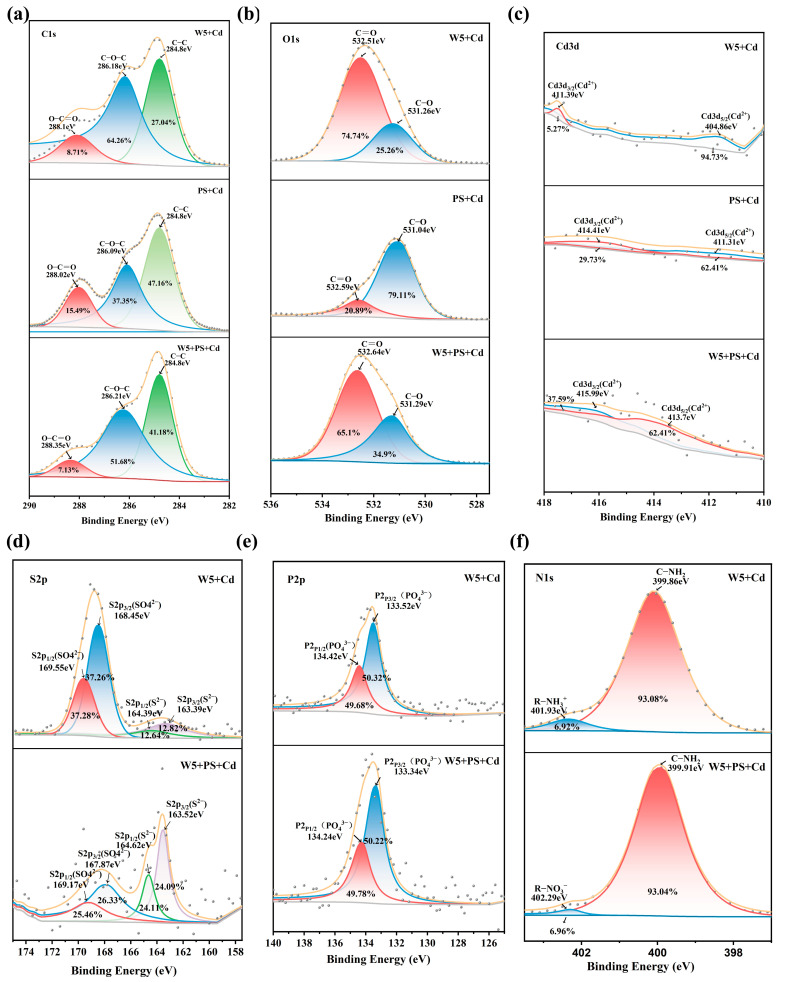
XPS spectra showing the co-immobilization of Cd by strain W5 and PS. High-resolution scans of (**a**) C 1s, (**b**) O 1s, (**c**) Cd 3d, (**d**) S 2p, (**e**) P 2p, and (**f**) N 1s core levels. Gray scatter points: raw data; red, blue, green, and purple solid lines: peak-fitting results; yellow solid line: total fitting envelope; light gray solid line: background baseline.

SEM-EDS observations revealed that strain W5 can effectively colonize the surface of PS and adsorb Cd^2+^ (Figure 3), whereas no bacterial attachment was seen on PS alone or PS + Cd (Figure S1a,b). Cd was detected on both bacterial cells (Figure S1c) and PS surfaces (Figure S1b and Figure 3). This supports the ability of strain W5 to colonize PS and participate in Cd immobilization. Consistently, the biofilm biomass of strain W5 on PS reached an OD_570_ of 1.67 ± 0.31 (*n* = 3) under the same Cd-PS combined stress (Table S3). Changes in surface elements and functional groups were further analyzed using X-ray photoelectron spectroscopy (XPS) (Figure 4). In the W5 + Cd treatment group, the contents of C–O–C and C=O were relatively high, at 64.26% and 74.74%, respectively, which is consistent with the possibility that the strain may adsorb Cd^2+^ via hydroxyl and ether groups. In the PS + Cd treatment group, O–C=O and C–O increased significantly. However, pristine polystyrene does not contain carboxyl groups; the origin of these signals (for example, from the culture medium or minor surface oxidation) is unclear. In the W5 + PS + Cd treatment group, the contents of C–O–C and C=O decreased to 52.68% and 65.1%, respectively, compared to the W5 + Cd treatment. This change may reflect a different surface chemical environment when both components are present, but the exact nature of the interaction requires further investigation. Furthermore, in the W5 + PS + Cd treatment group, the content of S^2−^ increased to 24.09%, and XPS analysis revealed a decrease in C–NH_2_ moieties accompanied by an increase in R–NO_2_^−^ species, suggesting a change in the oxidation state of nitrogen-containing groups. Simultaneously, characteristic peaks of Cd3d appeared in the XPS spectra, confirming the successful immobilization of Cd on the surfaces of both the bacterial strain and PS. The splitting of the Cd 3d XPS peaks indicated the coexistence of multiple chemical forms of Cd. XRD analysis (Figure S2) showed characteristic peaks corresponding to CdS, CdO, CdCO_3_, and Cd_3_(PO_4_)_2_ based on comparison with ICDD PDF standards. Comparison with standard reference spectra revealed the presence of CdS and CdO on the surface of the bacterial biofilm, while CdCO_3_ and CdO were detected on the polystyrene surface. In the composite system (W5 + PS + Cd), the signal for Cd_3_(PO_4_)_2_ was significantly enhanced (Figure S2). These results suggest that the synergistic action between strain W5 and PS promotes the transformation of Cd into stable precipitates, thereby enhancing Cd immobilization. However, we note that definitive phase identification by XRD remains challenging due to the complex sample matrix and possible peak overlaps.

### 2.5. Strain W5 Alleviates Cd Stress by Regulating Wheat Cd Accumulation

**Figure 5:**
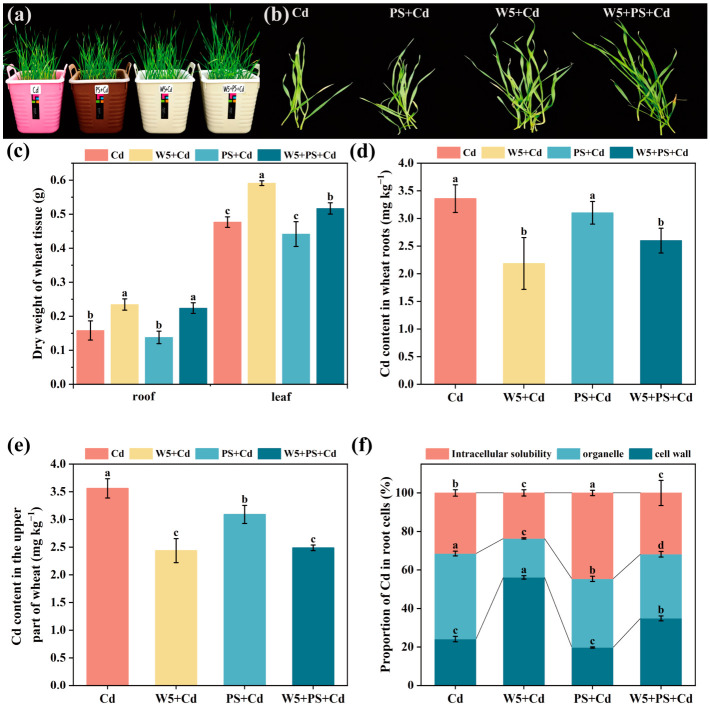
Effects of strain W5 on wheat growth and Cd accumulation under Cd-PS stress. (**a**,**b**) Phenotypic images; (**c**) Dry weight; (**d**) Cd content in roots; (**e**) Cd content in the upper part; (**f**) Subcellular distribution of Cd in roots. Error bars represent standard error (*n* = 3). Bars indicated by the same letter were not significantly (*p* > 0.05) different according to Tukey’s test.

Strain W5 alleviated the toxicity of Cd and Cd -PS combined stress on plants by regulating Cd accumulation and its subcellular distribution in wheat. Hydroponic experiments (Figure 5a–c and Figure S3) showed that Cd treatment alone significantly reduced wheat root length, root dry weight, and leaf dry weight by 7.8%, 21.0%, and 16.5%, respectively. In contrast, the addition of strain W5 increased plant height, root length, root dry weight, and leaf dry weight by 23.4%, 23.5%, 16.9%, and 16.5%, respectively. Under Cd-PS combined stress, inoculation with W5 still significantly enhanced plant height, root length, and root dry weight. Furthermore, strain W5 effectively reduced Cd accumulation in wheat tissues (Figure 5d,e). Compared to the Cd treatment group, the W5 + Cd treatment reduced Cd content in the shoots and roots by 31.6% and 34.9%, respectively. In the W5 + PS + Cd treatment, Cd content in the shoots and roots also decreased by 32.2% and 22.6%, respectively. An analysis of subcellular distribution revealed (Figure 5f) that in the Cd-treated group, Cd was primarily distributed in cell organelles and the intracellular soluble fraction, accounting for 44.2% and 31.4%, respectively. Following inoculation with strain W5, the proportion of Cd associated with the cell wall increased, while the proportions in the intracellular soluble fraction and organelles decreased. This indicates that W5 enhanced the cell wall’s capacity to immobilize Cd, reducing its translocation to active cellular components and thereby alleviating its toxic effects. Under the Cd-PS combined treatment, W5 was still able to induce a similar distribution trend, further confirming its role in enhancing wheat tolerance by modulating the subcellular compartmentalization of Cd.

### 2.6. Analysis of Differentially Expressed Proteins in Wheat Roots

**Figure 6:**
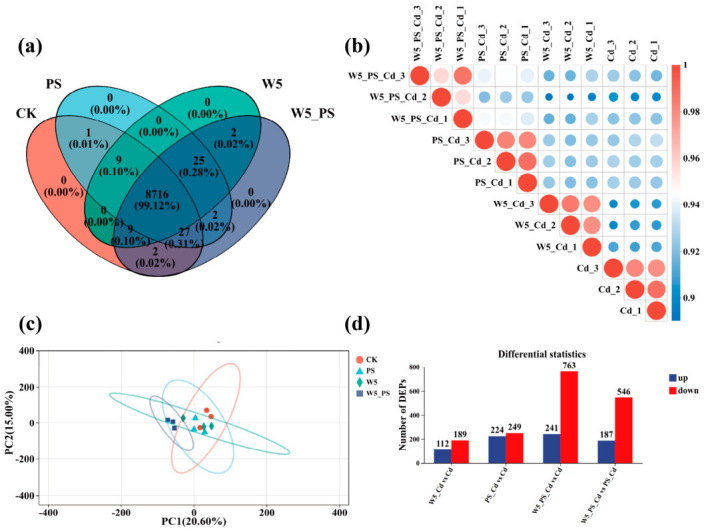
Analysis of distribution, correlation, principal components, and expression regulation characteristics of differentially expressed proteins (DEPs) in wheat roots. (**a**) Venn diagram; (**b**) Sample correlation heatmap; (**c**) PCA plot; The first two principal components (PC1, 20.60%; PC2, 15.00%) are shown. Symbols represent biological replicates (CK, PS, W5, W5_PS). Ellipses indicate 95% confidence ellipses (Hotelling’s T^2^, α = 0.05) for each treatment group. (**d**) Bar chart showing the numbers of up-regulated (red) and down-regulated (blue) DEPs under different treatment combinations.

Venn diagram analysis revealed that only 87 common differentially expressed proteins (DEPs) were identified across the different treatment groups, suggesting high specificity in the protein expression response of wheat roots under each treatment condition (Figure 6a). The sample correlation heatmap and principal component analysis (PCA) showed that replicate samples within groups clustered tightly with high correlations, while different treatment groups were clearly separated in three-dimensional space, thereby demonstrating good experimental reproducibility and significant differences in protein expression profiles among treatments (Figure 6b,c). PCA further showed that PC1 and PC2 explained 20.60% and 15.00% of the total variance, respectively, with samples from the same treatment clustering closely and distinct separation among groups. A statistical analysis of DEP numbers showed that the W5_PS_Cd vs. Cd group exhibited the highest number of DEPs, which were predominantly down-regulated; the W5_Cd vs. Cd group had fewer DEPs, indicating varying degrees of impact and regulatory tendencies on protein expression under different treatments (Figure 6d). The volcano plots further revealed (Figure S4a–d) that, compared to the single Cd treatment, the number of DEPs in the PS_Cd vs. Cd group increased to 473, with 224 up-regulated and 249 down-regulated. This indicates that PS altered the Cd-induced protein expression profile, resulting in additional differentially expressed proteins. Upon inoculation with strain W5, the number of DEPs in the W5_Cd vs. Cd group decreased to 301, which were predominantly down-regulated (189 proteins), suggesting that strain W5 may alleviate Cd toxicity by modulating protein expression. Under the combined pollution condition, the number of DEPs in the W5_PS_Cd vs. PS_Cd group reached 733, with 546 of them being down-regulated, demonstrating that strain W5 could reverse the expression abnormalities synergistically triggered by PS and Cd. Compared to the single Cd treatment, the W5_PS_Cd vs. Cd group exhibited 1004 DEPs, with down-regulated proteins constituting 76% (763 proteins). This suggests that strain W5 may induce a reorganized physiological state in response to combined stress through the extensive reprogramming of the proteome.

### 2.7. Enrichment Analysis of DEPs

**Figure 7:**
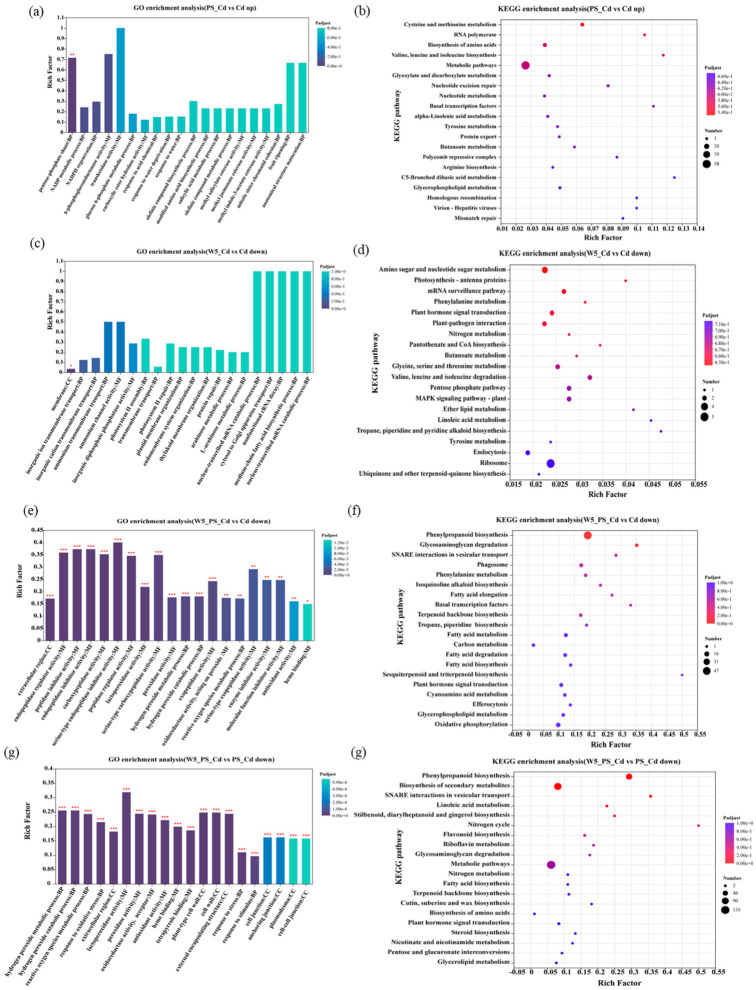
GO and KEGG enrichment analysis of DEPs in the underground parts of wheat roots under different treatment conditions. (**a**) GO (PS_Cd vs. Cd, up); (**b**) KEGG (PS_Cd vs. Cd, up); (**c**) GO (W5_Cd vs. Cd, down); (**d**) KEGG (W5_Cd vs. Cd, down); (**e**) GO (W5_PS_Cd vs. PS_Cd, down); (**f**) KEGG (W5_PS_Cd vs. PS_Cd, down); (**g**) GO (W5_PS_Cd vs. Cd, down); (**h**) KEGG (W5_PS_Cd vs. PS_Cd, down). In the GO plots, the color intensity of the bars reflects the significance of enrichment, with darker shades indicating a higher significance level. The color gradient of the bars represents the significance of enrichment. Bars with P or FDR < 0.001 are marked with ***, those with P or FDR < 0.01 are marked with **, and those with P or FDR < 0.05 are marked with *. In the KEGG plots, the size of the circles represents the number of DEPs enriched in the pathway. A larger circle indicates a greater number. The color of the circles represents the *p*-value from the hypergeometric test. A *p*-value closer to 0 (represented by red) indicates a more significant enrichment.

To elucidate the functional characteristics of the DEPs, Gene Ontology (GO) and Kyoto Encyclopedia of Genes and Genomes (KEGG) enrichment analyses were performed on the DEPs identified in wheat roots under Cd stress, PS stress, and their combined stress. GO enrichment analysis revealed that in the PS_Cd vs. Cd group, up-regulated proteins were primarily involved in processes such as the pentose phosphate pathway and NADP metabolism (Figure 7a); this pathway generates NADPH for glutathione regeneration, thus countering oxidative stress. In the W5_Cd vs. Cd group, down-regulated proteins were significantly enriched in functions related to ion transmembrane transport (Figure 7c); the downregulation of ion transporters may limit Cd influx. In the W5_PS_Cd vs. PS_Cd group, down-regulated proteins were mainly associated with pathways such as oxidative stress response and reactive oxygen species metabolism (Figure 7e); their suppression indicates reduced oxidative burden in the presence of W5. In the W5_PS_Cd vs. Cd group, differential proteins were predominantly linked to molecular functions including endopeptidase inhibition and oxidoreductase activity (Figure 7g)*,* which protect against proteolysis and maintain redox balance. KEGG enrichment analysis further revealed that up-regulated proteins in the PS_Cd vs. Cd group were significantly enriched in cysteine and methionine metabolism (Figure 7b); cysteine is a precursor of glutathione and phytochelatins for Cd chelation. Down-regulated proteins in the W5_Cd vs. Cd group were primarily concentrated in amino sugar and nucleotide sugar metabolism pathways (Figure 7d); these pathways are linked to cell wall synthesis; their downregulation may reflect resource reallocation. Down-regulated proteins in the W5_PS_Cd vs. PS_Cd group were notably enriched in pathways such as phenylpropanoid biosynthesis, flavonoid synthesis, and nitrogen metabolism (Figure 7f); phenylpropanoids and flavonoids chelate Cd and scavenge ROS, while nitrogen metabolism changes may affect Cd uptake. Down-regulated proteins in the W5_PS_Cd vs. Cd group were mainly associated with phenylpropanoid synthesis, oxidative phosphorylation, and fatty acid metabolism (Figure 7h); the downregulation of oxidative phosphorylation may reduce mitochondrial ROS production. These results indicate that different stress treatments induced specific responses in the functional and metabolic pathways of the wheat root proteome. This provides an important basis for elucidating the molecular mechanisms by which strain W5 alleviates combined stress.

### 2.8. Analysis of Commonly Downregulated Proteins

**Figure 8:**
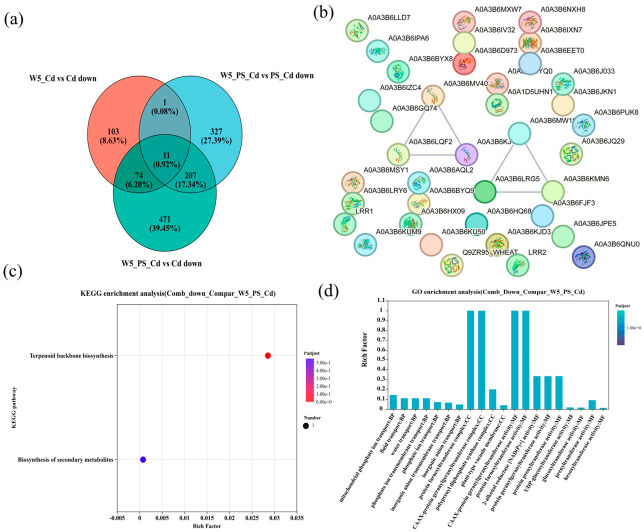
GO and KEGG enrichment analyses of 11 common differentially expressed proteins across the W5_Cd, W5_PS_Cd, and PS_Cd treatment groups. (**a**) Venn diagram; (**b**) Protein–protein interaction network; (**c**) GO enrichment; (**d**) KEGG pathway enrichment.

An integrated analysis of the down-regulated proteins across the three treatment groups—W5_Cd, W5_PS_Cd, and PS_Cd—identified a set of 11 commonly regulated DEPs (Figure 8a). These proteins were consistently down-regulated in all three groups. Protein–protein interaction network analysis further indicated close interactions among them, thus suggesting that they may function as a synergistic unit in the stress response (Figure 8b). The GO enrichment results indicated that these proteins are primarily involved in biological processes such as metabolic process and cellular catabolic process (Figure 8c). KEGG analysis further revealed that these proteins are significantly enriched in the biosynthesis of secondary metabolites pathway (Figure 8d). This suggests that the coordinated downregulation of these secondary metabolism-related proteins may enhance plant stress adaptability by coordinately down-regulating the expression of proteins related to secondary metabolism, thereby reallocating cellular resources to detoxification and fundamental maintenance processes.

### 2.9. qRT–PCR Verification

Based on the functional annotation and enrichment analysis of differentially expressed proteins (DEPs) in wheat roots, four Cd-responsive DEPs were selected for gene expression analysis using quantitative real-time PCR (qRT-PCR): ABC transporter (ABCC), glutathione S-transferase (GST), 12-oxophytodienoate reductase (OPR), and chalcone synthase (CHS) (Table S4). The results demonstrated that the expression patterns of these genes were consistent with those observed in the label-free proteomic data. For instance, the expression levels of ABCC and GST in the W5 + Cd treatment group were 1.3 and 2.1-fold higher than those in the Cd treatment group, respectively (Figure S5). Similarly, OPR and CHS showed downregulation under combined stress, which is consistent with the proteomic trends (Figure S5). Overall, the trends in gene and protein expression were similar, which validates our findings.

## 3. Discussion

### 3.1. Colonization and Synergistic Cd Immobilization Mechanism of Biofilm-Forming Bacterium W5 in a Cd-PS Combined System

Heavy metal-immobilizing bacteria primarily achieve metal fixation through biosorption, bioprecipitation, and redox reactions [18,19,20]. Among these, biofilm-forming bacteria secrete EPS rich in functional groups such as carboxyl, hydroxyl, and amino moieties, which can efficiently bind heavy metal ions via complexation, ion exchange, and electrostatic adsorption [21,22,23]. Microplastic surfaces provide a unique ecological niche for microbial colonization, thus leading to the formation of the plastisphere [24]. In this study, Enterobacter sp. W5, which is a highly efficient biofilm-producing bacterium isolated from heavy metal-contaminated soil, exhibited a remarkable capacity for Cd immobilization (51.3%) in the Cd-PS combined system and demonstrated effective colonization on PS surfaces. Cheng [25] reported that biofilm colonization confers additional high-energy adsorption sites on microplastics, thereby significantly enhancing their heavy metal adsorption capacity. Using nanoscale secondary ion mass spectrometry (NanoSIMS), Zhou [26] provided the first visual evidence of the co-localization of biofilms and copper (Cu) on microplastic surfaces, demonstrating that the presence of biofilms substantially enhances Cu(II) adsorption. Furthermore, Yang [27] conducted an in situ exposure experiment in the mangrove ecosystem of Hainan Island and revealed that biofilms colonizing microplastic surfaces act as complex vectors and dynamic interfaces, thus significantly amplifying the adsorption and enrichment capacity for both heavy metals and organic pollutants.

Consistent with the roles of biofilms described above, upon the colonization of microplastic surfaces by strain W5, the functional groups within its EPSs may directly participate in the complexation and immobilization of Cd. Furthermore, bacterial metabolic activities such as phosphate solubilization and hydrogen sulfide (H_2_S) production can induce the formation of stable metal precipitates, thereby enhancing the synergistic immobilization of heavy metals [28,29,30]. However, it is important to note that polystyrene itself possesses independent Cd adsorption capacity due to its aromatic ring structure and surface functional groups including O–C=O, which may contribute to Cd removal even in the absence of bacteria. Therefore, the Cd immobilization observed in the W5 + PS + Cd system likely results from both microbial activity (biofilm-mediated complexation and precipitation) and abiotic PS adsorption. The present study demonstrated that following the colonization of strain W5 on PS surfaces, bacterial surface functional groups such as C–O–C and C=O acted synergistically with the O–C=O groups of PS to adsorb and induce the formation of CdS, CdO, and Cd_3_(PO_4_)_2_ precipitates. Consistent with these findings, Li [31] reported that the C=O peak of protein amide I, which is derived from tryptophan-containing substances in the loosely bound EPSs (LB-EPS) of Enterobacter sp. FM-1, was primarily responsible for Pb^2+^ complexation. Similarly, Huang [32] demonstrated that the phosphate-solubilizing bacterium Enterobacter sp. PMB-5 induced the formation of Cd_3_(PO_4_)_2_ precipitates.

Enterobacter species are ubiquitous in soil and water environments, and some strains have been reported as opportunistic pathogens or carriers of antibiotic resistance genes (ARGs) [33,34]. In this study, strain W5 exhibited resistance to gentamicin and tetracycline. Although the strain was isolated from Cd-contaminated soil and shows promise for bioremediation, its potential environmental release risks should not be overlooked. Future studies must conduct comprehensive safety assessments, including whole-genome sequencing to identify ARGs and virulence factors, as well as targeting non-target organisms such as earthworms, before any field application [35]. The development of ARG-free or biofilm-deficient mutants could be a strategy to mitigate biosecurity concerns while retaining Cd immobilization capacity. Collectively, these observations establish a foundation for the potential application of strain W5 in mitigating Cd uptake by wheat under Cd-PS combined pollution conditions, but with necessary precautions.

### 3.2. Mitigative Effect of Biofilm-Forming Bacterium W5 on Cd Accumulation in Wheat

The root system serves as the primary organ for Cd uptake and accumulation in plants, with the root cell wall acting as the initial barrier against Cd entry into cells. Its immobilization capacity directly influences Cd transcellular migration and long-distance translocation [36,37]. Subcellular distribution analysis revealed the physiological mechanism by which bacterium W5 mitigates Cd transport. In the Cd-only treatment group, Cd was predominantly distributed in the organelles (44.2%) and soluble fractions (31.4%); however, following W5 inoculation, the proportion of Cd retained in the cell wall increased significantly, with corresponding decreases in the soluble and organelle fractions (Figure 5f). This suggests that W5 enhances the Cd immobilization capacity of the cell wall, sequestering more Cd within the root cell wall and thereby reducing its translocation to aboveground tissues. However, direct evidence including cell wall Cd speciation or transporter activity is still needed to confirm this mechanism. Cao [38] investigated perennial ryegrass and found that low-Cd-accumulating cultivars primarily resist heavy metal stress by binding more Cd and As to the cell wall, accompanied by cell wall thickening; in contrast, high-accumulating cultivars store Cd and As in the soluble fraction. Zheng [39] reported that foliar application of zinc and zinc/selenium mixed solutions enhanced the binding efficiency between pectin in rice flag leaves and Cd, strengthening Cd immobilization in the cell wall and consequently reducing grain Cd accumulation. These findings suggest that enhancing the Cd retention capacity of the cell wall represents a conserved detoxification strategy in wheat under heavy metal stress.

### 3.3. Proteomic Analysis of Wheat Response Regulated by Strain W5 Under Combined Cd-PS Stress

Proteins are the direct executors of life activities, and the dynamic changes in their expression levels profoundly influence the response strategies of plants to environmental stress. Proteomics technologies enable the systematic analysis of dynamic changes in protein abundance, modification states, and interaction networks under stress conditions, thereby providing a basis for elucidating the molecular regulatory mechanisms of the biofilm-producing strain W5 [40,41]. In the PS_Cd vs. Cd group, upregulated proteins were primarily enriched in cysteine and methionine metabolism pathways (Figure 7b), suggesting that PS may exacerbate Cd toxicity by severely disrupting amino acid biosynthesis and metabolic pathways. Zhao [42] reported that small-sized polystyrene microplastics promote the synthesis and secretion of rhizosphere amino acids, thereby enhancing Cd uptake by maize roots. The interference of PS with amino acid metabolism may be related to changes in the rhizosphere microenvironment resulting from alterations in its surface properties.

GO enrichment analysis revealed that downregulated proteins in the W5_Cd vs. Cd group were significantly enriched in ion transmembrane transport functions (Figure 7c), while KEGG analysis indicated that these proteins were primarily involved in amino sugar and nucleotide sugar metabolism pathways (Figure 7d). This suggests that strain W5 may trigger a global reprogramming of wheat root proteome, potentially redirecting cellular resources toward stress adaptation; however, the specific regulatory mechanisms remain unclear. Wang [43] demonstrated through proteomic and metabolomic analyses of tobacco protoplasts that under Cd stress, plants rapidly supply energy via glycolysis and the TCA cycle while enhancing stress protein synthesis to alleviate oxidative damage. The GO enrichment analysis of the W5_PS_Cd vs. PS_Cd group revealed that downregulated proteins were primarily involved in processes such as response to oxidative stress and reactive oxygen species metabolism (Figure 7e). KEGG analysis indicated that these proteins were significantly enriched in phenylpropanoid biosynthesis, flavonoid biosynthesis, and nitrogen cycling pathways (Figure 7f). These enrichment results indicate that the downregulated proteins are mainly involved in secondary metabolism and stress-related pathways, implying that strain W5 may modulate these processes; the exact regulatory cascade needs further validation. This is consistent with the energy reallocation strategy reported by [44] in plants responding to heavy metal stress, whereby plants suppress certain secondary metabolic processes to prioritize energy utilization for fundamental detoxification and maintenance mechanisms. The GO analysis of the W5_PS_Cd vs. Cd group showed that differentially expressed proteins were mainly involved in functions such as endopeptidase inhibitor activity and oxidoreductase activity (Figure 7g), with KEGG enrichment in phenylpropanoid biosynthesis, oxidative phosphorylation, and fatty acid metabolism pathways (Figure 7h). This suggests that following the introduction of polystyrene to form combined pollution with Cd stress, strain W5 triggers global metabolic reprogramming to synergistically counteract physical adsorption and chemical toxicity, thereby suppressing endogenous reactive oxygen species (ROS) generation during metabolic processes and assisting wheat in establishing a new adaptive state. However, while proteomic enrichment suggests a potential reduction in endogenous ROS generation, direct physiological indicators including ROS content, antioxidant enzyme activities, and MDA levels were not measured. Therefore, conclusions regarding oxidative stress alleviation remain correlative and require experimental confirmation. Liu [45] reported similar findings in their study, demonstrating that plant growth-promoting bacteria (Bacillus sp. SL-413) significantly reduced ROS accumulation induced by combined microplastic and Cd pollution through upregulation of genes involved in phenylpropanoid biosynthesis, flavonoid biosynthesis, and related pathways, thereby alleviating oxidative stress in sorghum plants.

Through an integrated analysis of commonly downregulated proteins across the W5_Cd, W5_PS_Cd, and PS_Cd groups, a set of 11 core proteins was identified (Comb_Down_Compar_W5_PS_Cd) (Figure 8a). Based on sequence homology, the functions of several of these proteins are hypothetically associated with the wheat response to Cd stress, but their biological functions require further experimental validation. A0A3B6HQ68 is annotated as a potential metal-binding protein, suggesting a possible role in Cd^2+^ chelation; however, this function has not been confirmed experimentally. Li [46] investigated the hyperaccumulator Sedum plumbizincicola and found that the defensin gene SpPDF exhibits Cd chelation activity. The overexpression of this gene significantly increased Cd accumulation in roots while reducing its translocation to aboveground tissues. A0A3B6KJV9 is predicted to function in intracellular transport, which may be linked to the vacuolar sequestration of Cd chelates; this hypothesis remains to be tested. Liu [47] identified the metal tolerance protein OsMTP11 in rice, which localizes to the tonoplast of vascular bundle cells in leaves. Through interaction with the vacuolar sorting receptor OsVSR2, OsMTP11 transports Cd from the trans-Golgi network to prevacuolar compartments and ultimately sequesters it within vacuoles. Rice varieties carrying the superior allele exhibited significantly reduced grain Cd accumulation. A0A3B6HX09 (putative chaperone) and A0A3B6PUK8 (putative protease) are annotated as stress-related proteins, implying potential involvement in maintaining protein homeostasis under Cd-PS stress; their actual roles need independent verification. Boukadida [48] reported that under Cd stress, the digestive gland of mussels exhibited the coordinated upregulation of genes encoding chaperones involved in protein folding (HSP90, HSP70, HSP27, HSP26, and FKBP) and metal detoxification proteins (metallothioneins MT-10 and MT-20), thus reflecting the central role of the chaperone network in responding to Cd-induced protein misfolding and oxidative damage. Several proteins, including A0A3B6IZC4, are associated with secondary metabolic pathways, which is consistent with the KEGG enrichment results. Xin [49] studied Pontederia cordata and found that the phenylpropanoid pathway functions as a chemical defense in leaves in response to Cd^2+^ stress, with flavonoid biosynthesis directly or indirectly exerting antioxidant effects. In our study, the downregulation of flavonoid biosynthesis could be interpreted in two non-mutually exclusive ways: it may represent an adaptive energy redistribution strategy, or it could reflect stress-induced metabolic impairment. Further analyses are needed to distinguish between these possibilities. The coordinated downregulation of these proteins, through remodeling secondary metabolic networks and reallocating cellular resources toward detoxification and core maintenance processes, assists wheat in establishing a new adaptive state under combined Cd-PS stress.

### 3.4. Summary and Future Perspectives

This study systematically investigated the effects and mechanisms of the biofilm-producing strain Enterobacter sp. W5 in mitigating Cd uptake by wheat under combined Cd-PS pollution. The main findings are as follows: (1) Strain W5 colonized the surface of PS microplastics and synergistically immobilized Cd with PS; (2) Strain W5 significantly reduced Cd accumulation in both the aboveground tissues and roots of wheat; (3) Proteomic analysis revealed a core set of downregulated proteins enriched in secondary metabolic pathways. The functions of key proteins including A0A3B6HQ68 and A0A3B6KJV9 are currently inferred solely from bioinformatic annotations and enrichment analysis, without independent experimental validation. Their potential roles in Cd translocation regulation remain speculative. However, this study has several limitations. First, all experiments were conducted under hydroponic conditions, and the field-scale performance of strain W5 needs further validation. Second, the proteomic results are interpreted based solely on predicted functional annotations and enrichment analysis, lacking independent biochemical or genetic validation. The specific roles and regulatory mechanisms of key proteins including A0A3B6HQ68 and A0A3B6KJV9 remain unclear and require future functional verification such as gene knockout, overexpression, or protein activity assays. Third, direct physiological indicators of Cd toxicity including ROS content, antioxidant enzyme activities, and chlorophyll content were not measured; therefore, conclusions regarding oxidative stress alleviation are based on proteomic inference and require experimental confirmation. Fourth, the independent abiotic contribution of PS adsorption to Cd removal was not quantitatively separated from microbial effects. Fifth, the ecological and biosecurity risks associated with the environmental release of antibiotic-resistant Enterobacter sp. W5 have not been assessed. Sixth, the hydroponic experiment did not include a true Cd-free and PS-free control group; therefore, the absolute effects of Cd on wheat growth cannot be directly quantified from the original dataset. However, because all treatment groups contained the same initial Cd concentration (3 mg L^−1^), the comparisons among Cd-containing groups (Cd, PS + Cd, W5 + Cd, W5 + PS + Cd) remain valid for evaluating the relative effects of PS and strain W5 under Cd stress. Future studies should include full factorial controls to enable a complete assessment of both Cd and PS effects. Additionally, this study focused exclusively on PS microplastics; the generalizability of the findings to other microplastic types prevalent in the environment, such as PE, PP, and PVC, needs to be explored. Future research should integrate multi-omics approaches to decipher the spatiotemporal dynamics of the bacterium–microplastic–heavy metal interaction network, conduct functional validation of key proteins, and evaluate the practical remediation potential of strain W5 through field trials.

## 4. Materials and Methods

### 4.1. Screening of Biofilm-Forming Bacteria

Rhizosphere soil samples contaminated with Cd were collected from vegetable plants in the suburban area of Jiyuan City, Henan Province (35°03′ N, 112°61′ E). For bacterial isolation, 2 g of soil was suspended in 50 mL of sterile deionized water and shaken at 30 °C for 2 h. The suspension was serially diluted using a ten-fold gradient dilution method to obtain dilutions ranging from 10^−1^ to 10^−5^. Aliquots (0.2 mL) of each dilution were spread onto Congo red solid medium [50], which contained the following components per liter: 1.0 g of NaNO_3_, 1.2 g of Na_2_HPO_4_, 0.9 g of KH_2_PO_4_, 0.5 g of MgSO_4_, 0.5 g of KCl, 0.5 g of yeast extract, 0.5 g of acid-hydrolyzed casein, 0.2 g of Congo red, 5.0 g of cellulose powder, and 5.0 g of agar. The pH was also adjusted to 7.0. Inoculated plates were incubated at 30 °C for 3 days in a biochemical incubator (Shengwei-SPXD300, Shanghai, China). Colonies exhibiting a mucus-enriched surface morphology were selected and purified by repeated quadrant streaking until pure, single colonies were obtained. The isolates were assigned identification numbers and preserved for subsequent analyses. The basic physicochemical properties of the collected rhizosphere soil samples are shown in Table S5.

### 4.2. Evaluation of Cd Immobilization and Biofilm Formation

The strains were revived from glycerol stocks and cultured to the logarithmic growth phase. Bacterial suspensions were then inoculated at 2% (*v*/*v*) into 50 mL Erlenmeyer flasks containing LB liquid medium, supplemented with a Cd stock solution to achieve a final Cd concentration of 15 mg L^−1^. Each treatment was repeated three times [51,52]. The culture was grown in a thermostatic shaker at 30 °C and 180 rpm for 48 h. After cultivation, the culture was transferred to a 50 mL centrifuge tube, adjusted to OD_600_ = 1 (to normalize Cd removal efficiency to bacterial biomass), and then centrifuged at 4000 rpm for 5 min using a low-temperature centrifuge (PY180S, Huicheng, China). A 5 mL aliquot of the supernatant was collected, subjected to digestion, and the Cd concentration was determined using inductively coupled plasma optical emission spectrometry (ICP-OES, model ICPE-9820, Shimadzu, Japan). The Cd removal efficiency was subsequently calculated. The biofilm-forming capacity of the strains was assessed using the crystal violet staining method [53]. Bacterial suspensions from each strain in the logarithmic growth phase were diluted with 1/2 strength LB liquid medium to an optical density (OD_600_) of 1.0. Aliquots of 200 μL were transferred into a 96-well microtiter plate, with each strain tested in triplicate wells. Wells containing only sterile medium served as blank controls. The plate was then incubated statically at 30 °C for 72 h. Following incubation, the medium from each well was carefully aspirated. Non-adherent planktonic cells were removed by gently washing each well three times with sterile phosphate-buffered saline (PBS). The plates were subsequently air-dried at room temperature. To fix the adherent biofilm, 200 μL of methanol was added to each well for 20 min. After removing the fixative, the wells were washed three times with PBS and air-dried again. The biofilm was stained by adding 200 μL of a 0.1% (*w*/*v*) crystal violet solution to each well and incubating for 20 min at room temperature, keeping it protected from light. The staining solution was then discarded, and the wells were rinsed three times with PBS to remove excess dye. Following a final air-drying step, the bound crystal violet was solubilized by adding 200 μL of 33% (*v*/*v*) glacial acetic acid to each well and incubating for 20 min. The absorbance of the resulting solution in each well was measured at a wavelength of 570 nm using a microplate reader (Multiskan SkyHigh, Multiskan SkyHigh, Thermo Fisher Scientific, Waltham, MA, USA). Strains exhibiting both strong Cd removal and significant biofilm formation were selected for subsequent research.

### 4.3. Characterization of Biofilm-Producing Bacteria

#### 4.3.1. Cd Tolerance and Minimum Inhibitory Concentration (MIC)

A single colony of strain W5 from a slant culture was streaked onto Congo red solid medium supplemented with a gradient of Cd concentrations (0, 100, 200, 400, 600, 800, 1000, 1200 mg L^−1^). The plates were incubated at 30 °C for 3–5 days. Growth of the strain on a plate indicated tolerance to that Cd concentration. If no growth was observed at a given concentration, the strain was re-streaked from the original culture onto a fresh plate of the same Cd concentration (without inoculation from the original plate) for secondary verification. The highest Cd concentration at which no growth occurred after secondary verification was recorded as the minimum inhibitory concentration (MIC) for strain W5.

#### 4.3.2. Antibiotic Resistance

Resistance to gentamicin and tetracycline was assessed on Congo red solid medium supplemented with appropriate concentrations of each antibiotic, following the same streak-and-verify procedure.

#### 4.3.3. IAA and Siderophore Production

The ability of strain W5 to produce indole-3-acetic acid (IAA) was quantified via the Salkowski colorimetric method [54]. Siderophore production was determined using the chrome azurol S (CAS) overlay assay [55].

#### 4.3.4. Molecular Identification

Following preliminary identification by Gram staining, genomic DNA was extracted from strain W5. Species identification was performed through 16S rDNA gene sequencing [56].

### 4.4. Adsorption of Combined Polystyrene and Cd by Biofilm-Producing Bacteria

Target bacterial suspensions were inoculated at 2% (*v*/*v*) into 250 mL Erlenmeyer flasks containing 50 mL of LB liquid medium supplemented with Cd to achieve a final concentration of 15 mg L^−1^. The cultures were incubated at 28 °C with shaking at 180 r·min^−1^ for 7 days. The experiment included four treatments: Cd control (CK), Cd with polystyrene (PS), Cd with bacterial strain W5 (W5), and Cd with both PS and strain W5 (W5 + PS). Each treatment was performed in triplicate. Samples were collected at 0, 1, 3, 5, and 7 days. Each sample was centrifuged at 4000 r·min^−1^ for 10 min to obtain the supernatant. The Cd concentration in the supernatant was measured by inductively coupled plasma optical emission spectrometry (ICP-OES), and its pH value was determined using a pH meter (PHS-3CT, Jingsheng, Shanghai, China). Additionally, an aliquot of the culture broth was taken to measure its optical density at 600 nm (OD_600_) using a UV-visible spectrophotometer (Model T6 New Century, Beijing Purkinje General Instrument Co., Ltd., Beijing, China). Cd removal efficiency was calculated as (C_i_ − C_t_)/C_i_ × 100%, where C_i_ is the initial Cd concentration and C_t_ is the residual concentration in the supernatant. The amount of Cd adsorbed was determined from the difference between initial and residual concentrations. For detailed information on polystyrene materials, see Table S6.

### 4.5. Mechanisms of Cd Adsorption by Biofilm-Forming Bacteria in the Presence of PS

The precipitates collected on the 7th day of the adsorption experiment were fixed with 15 mL of 2.5% (*v*/*v*) glutaraldehyde solution at 4 °C for 4 h. The fixed samples were then centrifuged at 5000 r·min^−1^ for 10 min. Subsequently, the pellets were subjected to gradient dehydration using 50%, 75%, 90%, and 100% ethanol solutions, with each step involving immersion at room temperature for 10 min followed by centrifugation to remove the supernatant [53]. The dehydrated precipitates were freeze-dried for 48 h to obtain dry samples. The morphology of the samples was characterized by field-emission scanning electron microscopy (SEM-EDS, Hitachi SU8010, Hitachi High-Technologies Corporation, Tokyo, Japan). The phase composition was analyzed using an X-ray diffractometer (XRD, D-MAX2500, Rigaku Corporation, Tokyo, Japan), and the surface elemental chemical states were characterized by X-ray photoelectron spectroscopy (XPS, Thermo Fisher Scientific K-Alpha, USA). The method for determining the biofilm biomass on the polystyrene surface is the same as that elucidated in Section 4.3. The PS microplastics were used as received (pristine, not aged or surface-modified), and FTIR confirmed that there were no carboxyl peaks before the experiment.

### 4.6. Hydroponic Experiment with Wheat

Plump wheat seeds were selected, surface-sterilized with 75% ethanol after washing them with sterile deionized water, and then germinated at 28 °C for 1 d. Twenty uniformly germinated seeds were transplanted into draining pots containing 1.0 kg of quartz sand. Each pot was placed over a container holding 1.8 L of Hoagland’s nutrient solution (excluding calcium nitrate) [57]. The hydroponic experiment included four treatments: (1) Cd: nutrient solution supplemented with 3 mg L^−1^ Cd; (2) W5 + Cd: inoculated with bacterial strain W5 and supplemented with 3 mg L^−1^ Cd; (3) PS + Cd: amended with 0.5% (*w*/*w*) PS and 3 mg L^−1^ Cd; (4) W5 + PS + Cd: simultaneously inoculated with strain W5 and amended with 0.5% PS and 3 mg L^−1^ Cd. Each treatment was replicated three times, with each replicate consisting of one pot containing 20 seedlings. After 10 days of cultivation, the corresponding treatments were sprayed with a Cd solution (3 mg L^−1^) and inoculated with a suspension of strain W5 (OD_600_ = 1.0, approximately 10^8^ CFU mL^−1^) via root immersion. The nutrient solution was replaced weekly, with the re-supplementation of the corresponding Cd, bacterial strain, or PS. All plants were cultivated in a greenhouse with the temperature maintained at 20 ± 3 °C.

The PS microplastic concentration of 100 mg L^−1^ and particle size of 25 μm were chosen based on environmental relevance: (1) PS concentrations up to 1700 particles kg^−1^ have been reported in contaminated soils [58]; (2) PS is among the most abundant polymer types in global agricultural soils [59]; and (3) particle sizes < 1 mm dominate soil microplastic pollution and are frequently used in hydroponic studies [60]. Thus, our experimental conditions are within environmentally relevant ranges for mechanistic investigations.

### 4.7. Determination of Cd Accumulation and Subcellular Distribution in Wheat

#### 4.7.1. Measurement of Wheat Biomass

Approximately 20 wheat plants from each treatment were harvested. After thoroughly washing them with deionized water, the roots were immersed in a 3.7 g·L^−1^ EDTA-2Na solution for 10 min to remove surface-adsorbed metal ions, followed by rinsing three times with deionized water. The plants were then separated into roots and shoots. Each part was labeled, dried in an oven at 80 °C until a constant weight was achieved, and subsequently weighed to determine the dry biomass. Plant height and root length were measured using a ruler.

#### 4.7.2. Quantification of Cd Content in Wheat Roots and Shoots

Dried plant samples (0.1 g) were weighed and placed into crucibles. A mixture of 4.5 mL of nitric acid, 1.5 mL of hydrochloric acid, and 2 mL of perchloric acid was added to each sample, and the crucibles were covered for digestion. Each treatment group included three replicates. After digestion was complete, Cd concentration was determined using ICP-OES.

#### 4.7.3. Determination of Cd Content in Wheat Subcellular Fractions

Fresh root tissue (0.5 g) was homogenized in 30 mL of extraction buffer (containing 250 mM sucrose, 50 mM Tris-HCl buffer at pH 7.8, and 1 mM dithioerythritol). The homogenate was centrifuged at 2000 rpm for 5 min, and the resulting pellet was collected as the cell wall fraction. The supernatant was then subjected to further centrifugation at 12,000 rpm for 60 min at 4 °C. The pellet from this step was designated as the organelle fraction, and the final supernatant was collected as the soluble cellular fraction. Each fraction was transferred to a crucible, dried, and digested. The Cd content in each fraction was subsequently quantified by ICP-OES [61]. Although marker enzyme assays were not performed, repeated washing (three times for the cell wall fraction, twice for the organelle fraction) and the use of low-speed centrifugation specifically for cell wall recovery are standard practices to ensure fraction purity [61]. The Cd distribution patterns in Figure 5 are considered acceptable for comparative analysis.

### 4.8. Label-Free Proteomic Analysis of Wheat Roots

The procedures for the protein extraction, quality control, and sequencing of the 12 root samples were conducted by Majorbio Bio-pharm Technology Co., Ltd. (Shanghai, China). The specific experimental workflow is described below.

#### 4.8.1. Protein Extraction and Quality Control

Wheat root samples were ground to a fine powder under liquid nitrogen. Total proteins were extracted using a phenol/methanol–ammonium acetate method following homogenization in protein extraction buffer. The resulting protein pellets were washed with pre-chilled acetone and subsequently dissolved in a lysis buffer containing 8 M urea, 1% SDS, and protease inhibitors. To enhance solubility, the samples were subjected to low-temperature ultrasonication. Protein concentration was determined using the Bicinchoninic Acid (BCA) assay. The integrity of the proteins and the reproducibility among replicates within each group were assessed by SDS-PAGE. All samples yielded a total protein amount exceeding 50 µg and exhibited clear electrophoretic bands, meeting the requirements for subsequent analysis. Proteins with missing values in more than 50% of samples were excluded from analysis. No imputation was performed; instead, statistical comparisons were conducted only on proteins with complete data in at least two of the three replicates per condition. This conservative approach minimizes false positives due to imputation.

After missing value filtering and normalization, differentially expressed proteins (DEPs) between two treatment groups were identified using Student’s *t*-test. To account for multiple hypothesis testing, the Benjamini–Hochberg false discovery rate (FDR) correction was applied. Proteins with an FDR-adjusted *p*-value (q-value) < 0.05 and an absolute fold change (|FC|) ≥ 1.5 were considered as significantly differentially expressed. The same criteria were used for all pairwise comparisons.

#### 4.8.2. Peptide Preparation

Aliquots containing 100 μg of protein from each sample were denatured in 100 mM triethylammonium bicarbonate (TEAB) buffer. The proteins were subsequently reduced with 10 mM Tris(2-carboxyethyl)phosphine (TCEP) at 37 °C for 60 min and alkylated with 40 mM iodoacetamide (IAM) in the dark at room temperature for 40 min. The samples were then digested overnight at 37 °C with sequencing-grade trypsin at an enzyme-to-substrate ratio of 1:50 (*w*/*w*).

#### 4.8.3. LC-MS Analysis

Following digestion, the resulting peptides were desalted, vacuum-dried, and quantified using a NanoDrop One spectrophotometer. An equal amount of peptides from each sample was loaded onto a nanoflow high-performance liquid chromatography system (Vanquish Neo, Thermo Fisher Scientific). Separation was performed on a homemade reverse-phase C18 column (100 μm i.d. × 15 cm length, 1.7 μm particle size). Mobile phase A consisted of 2% acetonitrile with 0.1% formic acid, and mobile phase B contained 80% acetonitrile with 0.1% formic acid. Peptides were eluted using a linear gradient over 14.4 min. Eluted peptides were ionized and analyzed in positive ion mode on a timsTOF HT mass spectrometer (Bruker Daltonics, Billerica, MA, USA). The ion source voltage was set at 1.6 kV. Data-independent acquisition (DIA) was performed using a pre-defined parallel accumulation–serial fragmentation (dia-PASEF) scan mode, covering specified m/z and ion mobility windows to comprehensively acquire fragment ion information from all detected peptides in an unbiased manner.

#### 4.8.4. Data Analysis

Raw MS data were processed using Spectronaut™ software version 19.7 (Biognosys AG, Schlieren, Switzerland) for protein identification and label-free quantification. Proteins with an absolute fold change (|FC|) > 1.5 and a *p*-value < 0.05 were defined as differentially expressed. Gene Ontology (GO) enrichment and Kyoto Encyclopedia of Genes and Genomes (KEGG) pathway analyses were subsequently performed on the differentially expressed proteins.

### 4.9. Quantitative Real-Time PCR (qRT–PCR) Verification

To validate the proteomic data, the expression levels of selected genes involved in Cd detoxification and stress responses, including ABCC (ABC transporter), GST (glutathione S-transferase), OPR (12-oxophytodienoate reductase), and CHS (chalcone synthase), were analyzed in wheat roots using quantitative real-time PCR (qPCR). Total RNA was extracted using the TaKaRa MiniBEST Plant RNA Extraction Kit (TaKaRa Bio Inc., Otsu, Shiga, Japan), and 1 μg of RNA was reverse-transcribed with the PrimeScript™ RT Reagent Kit with gDNA Eraser (TaKaRa, Japan). The qPCR reactions were performed on an ABI 7500 Real-Time PCR System (Applied Biosystems, USA) using SYBR^®^ Premix Ex Taq™ (TaKaRa, Japan). Gene-specific primers were designed using Primer Premier 5.0 software, and the sequences are listed in Table S4. Prior to expression analysis, primer efficiency was assessed via standard curves generated from serial dilutions of cDNA. All primer pairs showed amplification efficiencies between 92% and 106% (R^2^ > 0.99). GAPDH was used as the internal reference gene. Relative gene expression levels were calculated using the 2^-ΔΔCt^ method [62]. All samples were analyzed in three biological replicates.

### 4.10. Statistical Analysis

Statistical analysis was performed using SPSS software (version 18.0). Significant differences among treatments were assessed by one-way analysis of variance (ANOVA) followed by Tukey’s post hoc test, with a significance level set at *p* < 0.05. Figures were generated using Origin 2024, Avantage 2022, and Adobe Illustrator 2025.

## 5. Conclusions

A highly efficient biofilm-producing bacterial strain, Enterobacter sp. W5, was isolated from rhizosphere soil contaminated with heavy metals. Strain W5 exhibited a Cd removal rate of 46.3% and strong biofilm-forming capacity (OD_570_ = 5.05). It effectively colonized the surface of PS microplastics, where functional groups on the bacterial surface—such as C–O–C and C=O—may have acted synergistically with the O–C=O groups of PS, thus potentially contributing to Cd^2+^ and the formation of Cd-containing precipitates including CdS, CdO, and Cd_3_(PO_4_)_2_, as suggested by XPS and XRD analyses. Hydroponic experiments revealed that strain W5 alleviated the dual stress of Cd and PS in wheat, promoting shoot and root elongation, increasing root and leaf dry weight, and reducing Cd accumulation by 22.6% in roots and by 34.2% in aboveground tissues. Under Cd-PS stress, strain W5 enhanced the Cd retention capacity of the cell wall, which may limit Cd migration into active cellular compartments. Proteomic analysis identified a set of 11 consistently downregulated proteins including A0A3B6HQ68 and A0A3B6KJV9, which were enriched in secondary metabolite biosynthesis pathways. Bioinformatic analysis suggests these proteins may be associated with Cd translocation regulation, though their exact functions require independent experimental validation. These findings provide both a theoretical basis and a microbial resource for utilizing biofilm-forming bacteria to mitigate combined heavy metal–microplastic pollution and for breeding low-Cd-accumulating crops.

## Data Availability

The raw data supporting the conclusions of this article will be made available by the authors, without undue reservation.

## Supplementary Materials

The following supporting information can be downloaded at: https://www.mdpi.com/article/10.3390/plants15111698/s1, Figure S1: SEM-EDS images (a) PS alone; (b) PS + Cd; (c) W5 + Cd. Figure S2: XRD patterns illustrating the co-immobilization of Cd by strain W5 in conjunction with PS. Figure S3: Effects of strain W5 on wheat growth and Cd accumulation under Cd-PS Stress—plant height and root length. Error bars represent standard error (*n* = 3). Bars indicated by the same letter were not significantly (*p* > 0.05) different according to Tukey’s test. Figure S4: Volcano plots of DEPs in wheat roots under different treatment conditions. The *X*-axis represents the fold change relative to the control, and the *Y*-axis represents the significance of differential expression. Gray circles indicate proteins that are not significantly different (0.83 < log_2_ FC < 1.2, *p* < 0.05); red circles indicate up-regulated proteins (log_2_ FC > 1.2, *p* < 0.05); blue circles indicate down-regulated proteins (log_2_ FC < 0.83, *p* < 0.05). Figure S5: Gene expression levels were determined by qRT-PCR. (a) ABCC; (b) GST; (c) OPR; (d) CHS. Cd: wheat roots treated with 3 mg L^−1^ Cd; W5 + Cd: wheat roots treated with 3 mg L^−1^ Cd and inoculated with strain W5; PS + Cd: wheat roots treated with 3 mg L^−1^ Cd and inoculated with 0.5% (*w*/*w*) PS; W5 + PS + Cd: wheat roots treated with 3 mg L^−1^ Cd and inoculated with strain W5 and 0.5% (*w*/*w*) PS. Error bars represent standard error (n = 3). Bars followed by the same letter are not significantly different (*p* > 0.05) according to Tukey’s test. Primer efficiencies ranged from 92% to 106% (see Materials and Methods for details). Table S1. The growth-promoting characteristics and resistance to heavy metals and antibiotics of strain W5. Table S2. The ability of the test strain to produce IAA and siderophores. Table S3. Biofilm biomass of strain W5 on polystyrene surfaces under combined Cd-PS stress. Table S4. Primer sequences for real-time PCR. Table S5. Physicochemical properties of contaminated soil used for the isolation of biofilm bacteria. Table S6. Basic Physicochemical Properties of Polystyrene.
