# Supplementary material for: Biofilm-Forming Enterobacter sp. W5 Mitigates Cadmium and Polystyrene Microplastic Stress in Wheat via Synergistic Immobilization and Proteomic Reprogramming
Source: Plants (Basel). 2026 May 30;15(11):1698. doi: 10.3390/plants15111698 (PMC13258922; doi:10.3390/plants15111698)
Supplement: Supplementary file 1 [file plants-15-01698-s001.zip › plants-4319604-supplementary.pdf]

Supporting Information for

Biofilm-Forming Enterobacter sp. W5 Mitigates Cadmium and Polystyrene Microplastic Stress in Wheat via Synergistic Immobilization and Proteomic Reprogramming

Jiexun Wang [1], Yun Li [1], Hao Zhang [1,2], Wenxia Wang [1,3], Lunguang Yao [2,3], Randa S. Makar [2,3], Zhaojin Chen [1] and Hui Han [1,3,*]

[1] Henan Key Laboratory of Ecological Security for Water Source Region of Mid-line of South-to-North Diversion Project, College of Water Resources and Modern Agriculture, Nanyang Normal University, Nanyang 473061, China; wangjiexun0416@163.com (J.W.); ly0210252026@163.com (Y.L.); zhanghao660@nynu.edu.cn (H.Z.); wenxiawang@nynu.edu.cn (W.W.); zhaojin_chen@163.com (Z.C.)

[2] Soils and Water Use Department, Agricultural and Biological Research Institute, National Research Centre, Dokki, Cairo 12622, Egypt; lunguangyao@nynu.edu.cn (L.Y.); randa_sgmm@yahoo.com (R.S.M.)

[3] Henan International Joint Laboratory of Soil Health and Water Security, Nanyang Normal University, Nanyang 473061, China

* Correspondence: 17657311626@163.com

Table S1. The growth-promoting characteristics and resistance to heavy metals and antibiotics of strain W5.

	W5
IAA(mg L^{-1})	65.4
Siderophore	++++
Lethal concentration of Cd (mg L^{-1})	800
Gram stain	-
Kanamycin	-
Gentamicin	+
tetracycline	+
streptomycin	-

Note: ++++represents extremely strong activity. (Siderophore Activity)

Table S2. The ability of the test strain to produce IAA and siderophores.

Strain	IAA(mg L^{-1})	Siderophore
W1	27.6	++
W2	26.48	+
W3	43.7	+++
W4	25.67	++
W5	65.4	++++
W6	56.74	++
W7	38.65	+
J1	51.97	+++
J2	58.8	++
J3	36.78	+
J4	50.71	++
J5	58.48	+++
X1	32.4	+
X2	27.6	+
X3	42.8	++

Note: +represents weak activity, ++represents strong activity, +++represents very strong activity, ++++represents extremely strong activity.

Table S3. Biofilm biomass of strain W5 on polystyrene surfaces under combined Cd-PS stress.

Replicate	OD_{570}
1	1.4
2	1.6
3	2.0
Mean ± SD	1.67 ± 0.31

Note: Biofilm biomass was determined by crystal violet staining (OD_{570}) after 7 d incubation of strain W5 with PS (25 μm) and Cd (15 mg L^{-1}). Data are three independent replicates (mean ± SD). See Materials and Methods for detailed procedure.

Table S4. Primer sequences for real-time PCR.

Gene ID	Primers	Sequence (5’→3’)
ABCC	ABCC-F	GGCTCGTTGTTGTTGGTTCTTATG
	ABCC-R	CCTCTTATCGGTCCGTTGTATGC
GST	GST-F	AAGAGATTGTCGGTGTCAGTTTGG
	GST-R	CGGCGTGTCCTGTTTAGAGAATAG
OPR	OPR-F	ACAGGGAGGAAGGGAACAAGG
	OPR-R	TTGGGCAGGTCAGGGTTAGC
CHS	CHS-F	CACCGTGGAGGAGTATCGTAAGGC
	CHS-R	TGATCAACACAGTTGGAAGGCG

Table S5. Physicochemical properties of contaminated soil used for the isolation of biofilm bacteria.

pH	organic matter (mg kg^{-1})	Total Cd (mg kg^{-1})
7.68	28.4	3.84

Table S6. Basic Physicochemical Properties of Polystyrene.

Manufacturer	Side(μm)	morphology	Water Contact Angle (°)	Specific surface area (m^2 g^{-1})
Suzhou Nawei Technology	20	granular	112.5	1.19

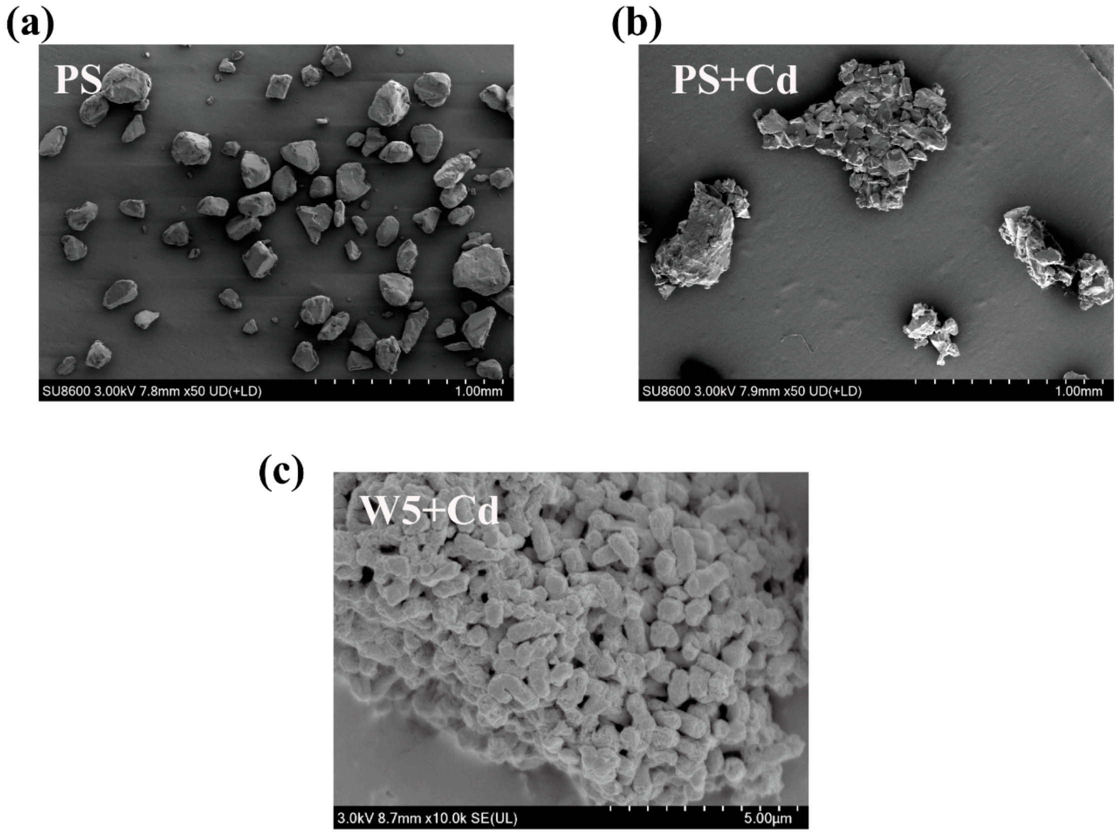

Figure S1. SEM-EDS images (a) PS alone; (b) PS+Cd; (c) W5+Cd.

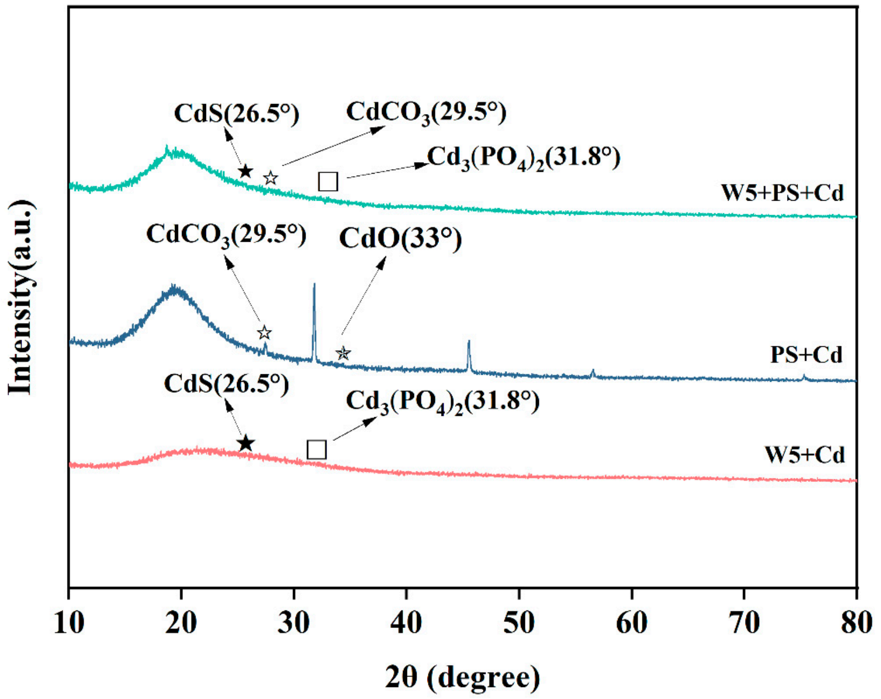

Figure S2. XRD patterns illustrating the co-immobilization of Cd by strain W5 in conjunction with PS. PDF#41-1049 for CdS, PDF#05-0640 for CdO, PDF#42-1342 for $CdCO_3$, PDF#31-0246 for $Cd_3(PO_4)_2$

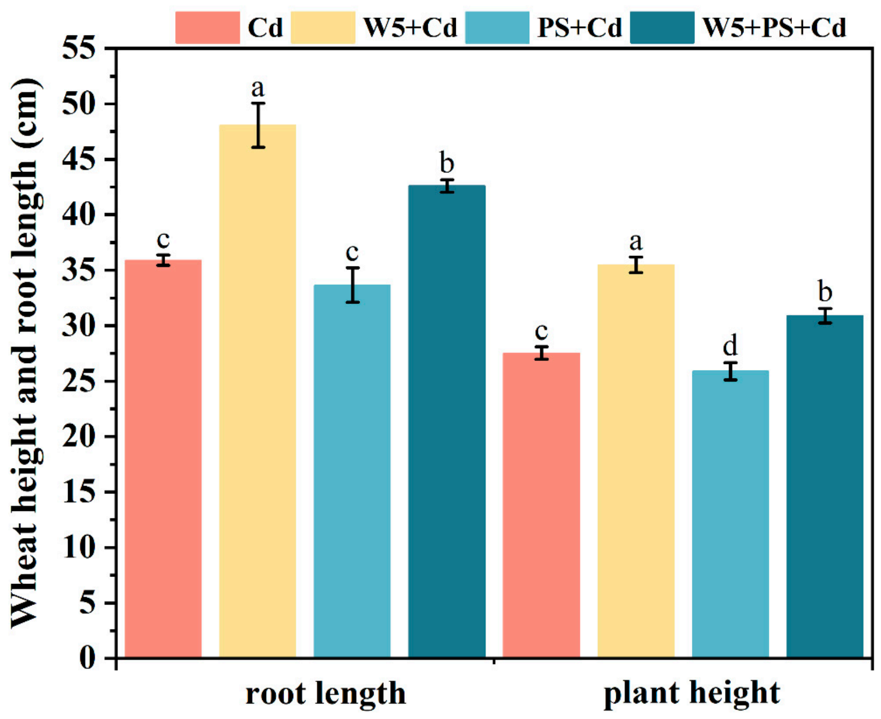

Figure S3. Effects of strain W5 on wheat growth and Cd accumulation under Cd-PS Stress — plant height and root length. Error bars represent standard error (n = 3). Bars indicated by the same letter were not significantly ($P > 0.05$) different according to Tukey's test.

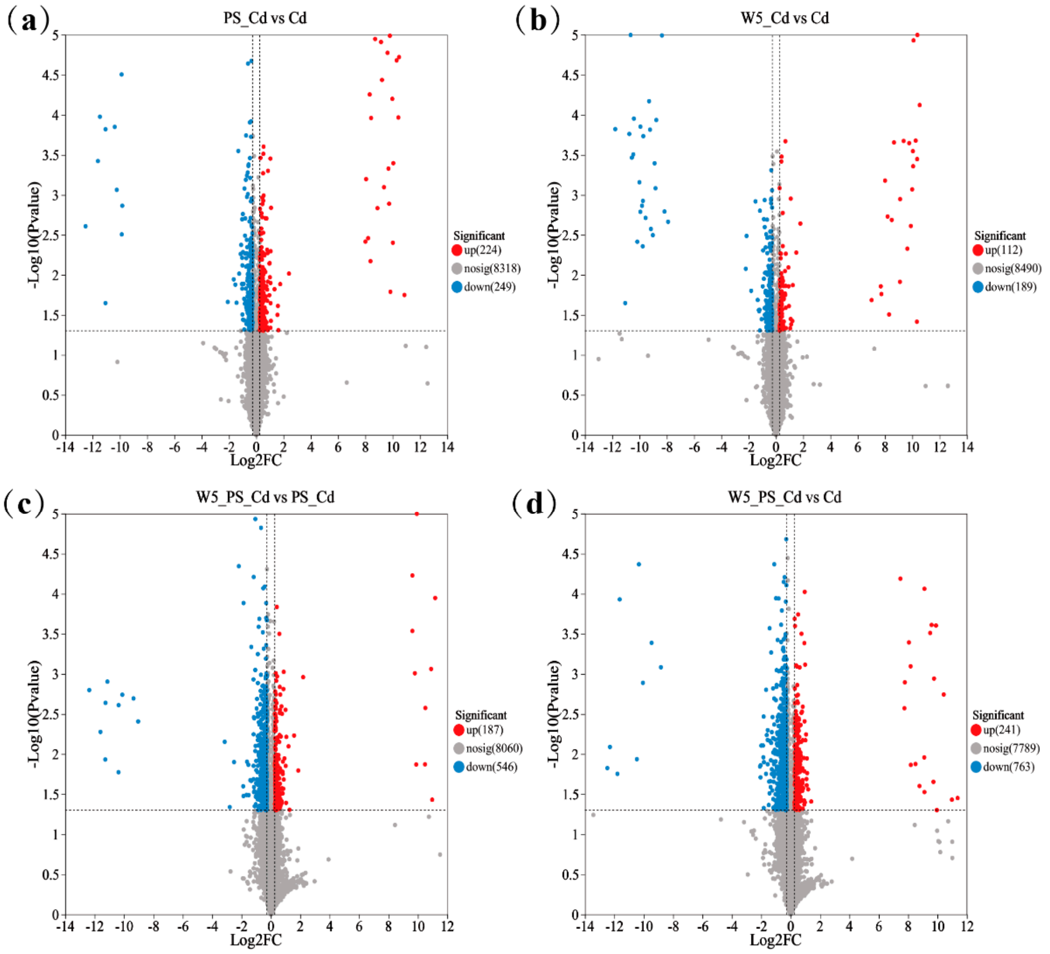

Figure S4. Volcano plots of DEPs in wheat roots under different treatment conditions. The X-axis represents the fold change relative to the control, and the Y-axis represents the significance of differential expression. Gray circles indicate proteins that are not significantly different ($0.83 < \log_2 FC < 1.2$, $P < 0.05$); red circles indicate up-regulated proteins ($\log_2 FC > 1.2$, $P < 0.05$); blue circles indicate down-regulated proteins ($\log_2 FC < 0.83$, $P < 0.05$).

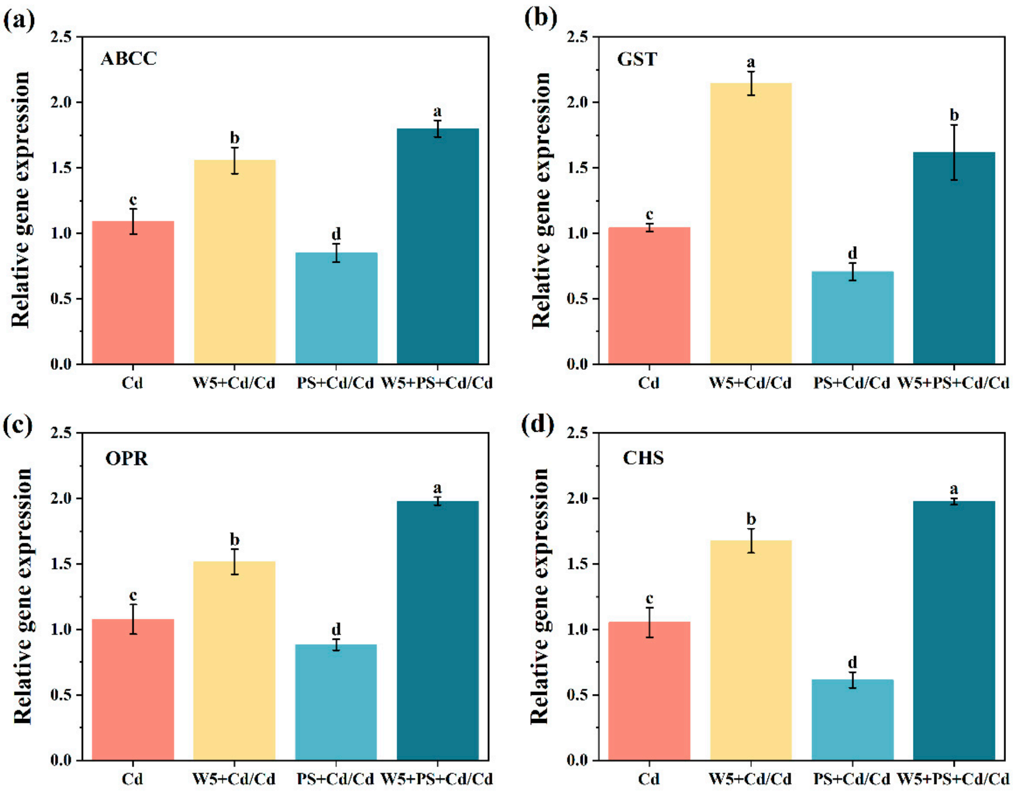

Figure S5. Gene expression levels were determined by qRT-PCR. (a) ABCC; (b) GST; (c) OPR; (d) CHS. Cd: wheat roots treated with 3 mg L^{-1} Cd; W5+Cd: wheat roots treated with 3 mg L^{-1} Cd and inoculated with strain W5; PS+Cd: wheat roots treated with 3 mg L^{-1} Cd and inoculated with 0.5% (w/w) PS; W5+PS+Cd: wheat roots treated with 3 mg L^{-1} Cd and inoculated with strain W5 and 0.5% (w/w) PS. Error bars represent standard error (n = 3). Bars followed by the same letter are not significantly different ($P > 0.05$) according to Tukey's test. Primer efficiencies ranged from 92% to 106% (see Materials and Methods for details).
